# Perceived Object Continuity and Spontaneous Retrieval of Features from an Inhibited Object

**DOI:** 10.1371/journal.pone.0063264

**Published:** 2013-05-16

**Authors:** Zhe Chen, Yei-Yu Yeh

**Affiliations:** 1 Department of Psychology, University of Canterbury, Christchurch, New Zealand; 2 Department of Psychology, National Taiwan University, Taipei, Taiwan; University of Bath, United Kingdom

## Abstract

Previous research has shown that attention to an object can trigger the retrieval of features of a preceding object. The present study investigates whether such retrieval would occur to a recently inhibited object. In three experiments, participants saw two successively presented stimuli (S1 and S2) that varied in color and orientation. The task was to respond to the color or orientation of S2 in accordance with a task cue at the beginning of each trial. In separate experiments, we manipulated the number of the trials on which the task relevant features of S1 and S2 were matched versus mismatched, and the perceived object continuation between the two stimuli. Evidence for spontaneous feature retrieval was found when S1 and S2 could be seen as different instantiations of the same object but not when they were likely to be perceived as different types of objects. These results suggest that the features of a previously inhibited object can be retrieved spontaneously. However, such retrieval and its effect on a subsequent stimulus depend on the perceived object continuity between the two successive stimuli.

## Introduction

Visual perception occurs over time and space. In order to make sense of a continuous flow of information which is frequently occluded by other objects or interrupted by eye movements, our visual system must determine the relationship between successive stimuli. Given the prevalence of occlusion and saccades in visual perception, it is perhaps not surprising that our visual system can spontaneously retrieve features from a previously seen object when it is perceived as a different instantiation of a subsequent object [1]–[7]. However, so far, there has not been much research on the spontaneous retrieval of features of a recently inhibited object. The present study focuses on two issues: (1) whether features of a previously viewed object can be retrieved spontaneously when that object has recently been inhibited, and (2) whether such retrieval is also contingent upon the perceived object continuity between the two successive stimuli.

An influential theoretical framework that addresses the relationship between object continuity and visual information processing is the object file theory [6],[8]. According to this theory, when an object is encountered, the visual system creates an “object file”: a temporary episodic representation that contains information about the features of the object. Attention to an object triggers an automatic process of reviewing. When two stimuli appear in close spatiotemporal proximity, depending on their perceived object continuity, the reviewing process will lead to either the updating of a pre-existing object file or the creation of a new one. If an object link is found through a correspondence process between the current object, S2, and an object viewed recently, S1, then the two stimuli are seen as different states of the same object, and the contents of the previous object file are retrieved, and updated if necessary. However, if an object link is not found between S2 and S1, then the two stimuli are seen as belonging to different objects, and a new object file is created for S2. As updating an existing object file requires fewer mental resources than creating a new one, responses to S2 are facilitated when S1 and S2 are perceived as different states of the same object rather than as two different objects.

The object file theory has been supported by many studies [1],[2],[6],[7]. For example, in a series of experiments, Kahneman et al. showed participants two successive displays, a preview display with two or more letters, each in an individual frame, and a target display with a single letter in one of the frames. The task was to report the identity of the target letter. The main finding was an object-specific preview effect: responses to the target were reliably faster when it was a previewed letter that appeared in the same frame (absolute or relative) compared with a previewed letter that appeared in a different frame. Furthermore, the benefit of priming, i.e., facilitation due to prior exposure of a stimulus relative to a new stimulus, was small and unreliable. These results suggest that the object-specific preview effect in Kahneman et al.’s paradigm was derived primarily from the retrieval of the features of a previously encountered stimulus by a current object.

More recently, Hommel and his colleagues [3]–[5] extended the object file theory to include response-related information in the episodic representation of an attended object. Hommel coined the term “event file” to emphasize a multi-layered network of bindings among stimulus features, response features, and task context. The general idea of the theory is that the co-occurrence of stimulus features, or the co-occurrence of a stimulus feature and an action (e.g., a left or right response), causes them to bind spontaneously (but see Hommel [3] for evidence of binding between shape and location when shape was task relevant, but not when color was task relevant). Once they are bound, the activation of one leads to the activation of the other. Consequently, a partial match between S1 and S2 delays responses to S2 relative to both a complete match and a complete mismatch between the two stimuli. This partial-repetition cost is presumably caused by the extra time it takes to resolve the conflict induced by a previous binding [4],[5]. Thus, if participants respond to the onset of S1 (i.e., features of S1 are task irrelevant) but to the form of S2, repeating the form of S1 in S2 would produce a cost not only when the colors of S1 and S2 differed (a partial match) relative to when their colors matched (a complete match), but also when the colors of S1 and S2 differed (again a partial match) compared with when both the form and the color of S1 and S2 differed (a complete mismatch). This pattern of data is exactly what Hommel and his colleagues observed in many of their experiments [3],[5],[9].

If we assume that a complete match between the features of S1 and S2 would induce participants to see the two stimuli as different states of the same object, the above studies provide evidence for the spontaneous retrieval of features of S1 when object continuity is perceived between S1 and S2. What is less clear is whether the spontaneous retrieval of features would still occur when a previously viewed object has just been inhibited, and whether such retrieval would also be contingent upon the perceived object continuity between the two stimuli. If participants know that the chances of S1 being the same as S2 are small, will attention to S2 trigger the retrieval of S1 features in a way similar to that observed in previous studies where S1 was not suppressed [5],[9]? Such a mechanism would have the advantage of reducing the processing load of S2 when S1 and S2 are perceived as different instantiations of the same object. However, the same mechanism would not be particularly helpful when S1 and S2 belong to different types of objects, for the exact combination of features in S1 would never repeat in S2.

There is some indication in prior research that the spontaneous retrieval of features from an inhibited object can occur when it is identical to a subsequently presented target. Using a negative priming paradigm [10], Tipper, Weaver, and Houghton [11] manipulated the relationship between a distractor on a prime trial (trial n) and a target on a probe trial (trial n+1). In one experiment (Experiment 1), participants saw displays that consisted of two colored letters in two of four marked locations. One of the letters was a target, and the other was a distractor. The target was defined by color, and the task was to report the target’s location. On some trials (the control condition), the stimuli on the probe trial were unrelated to the stimuli on the prime trial. On other trials (the ignored repetition conditions), the target on the probe trial matched the distractor on the prime trial in one or more of its features (i.e., color, location, and/or form). Relative to the control condition, reaction times (RTs) to the probe target were longer in most conditions when the probe target had the same location and/or color as that (or those) of the prime distractor, demonstrating negative priming. Negative priming was not found when the prime and probe matched only in form, which was a task irrelevant feature. Furthermore, positive priming was found when the prime and probe were identical (i.e., a complete match in all the features of the prime and probe) compared with when they were unrelated or when there was only a partial match in their features. These results were interpreted by Tipper et al. [11] in terms of the flexibility of the visual system: a system that can have multiple levels of internal representation and can evoke task-specific inhibition. Their results are also consistent with the notion that the features of an inhibited object can be spontaneously retrieved if the inhibited object can be seen as a different instantiation of a subsequent object (cf [12]–[14] for non-inhibition interpretations of negative priming).

We were interested in the effect of inhibition on the retrieval of object features when a display consisted of a single object, and participants knew in advance that two successively presented objects were unlikely to be identical. In three experiments, participants saw a task cue, followed by S1 and then S2. Both S1 and S2 consisted of a two-dimensional stimulus that varied in color and orientation. The task was to respond to S2 while ignoring S1. In Experiment 1, S1 and S2 were independent. This experiment was conducted to ensure that spontaneous feature retrieval could occur in our paradigm when inhibition was not evoked. In Experiments 2 and 3, we matched the task relevant features of S1 and S2 on one-third of the trials, and mismatched them on the rest of the trials. We manipulated the identity of S2 so that S1 and S2 were likely to be seen as different instantiations of the same object in Experiment 2 but as different types of objects in Experiment 3. We found evidence for spontaneous feature retrieval in Experiments 1 and 2, but not in Experiment 3. Together, these results suggest that features of a previously inhibited object can be retrieved spontaneously, but such retrieval occurs only when the two stimuli are seen as different instantiations of the same object.

## Experiment 1

The goal of Experiment 1 was two-fold: to ensure that spontaneous feature retrieval could occur with changing behavioral goals, and to provide a baseline for Experiments 2 and 3. In prior research on feature retrieval, participants typically performed the same task from beginning to end in an experiment [1],[5],[6]. In the present experiment, they had to switch tasks from trial to trial on the basis of a task cue at the beginning of each trial. In experiments with a single task, the task irrelevant feature may become less salient over time. In contrast, when participants are required to switch between two tasks, the irrelevant feature is likely to be kept salient, and this in turn may influence object-specific feature retrieval. Thus, if evidence for spontaneous feature retrieval was found in Experiment 1, this would generalize the results of prior research to situations where participants’ behavioral goals changed constantly across trials.

On each trial, participants saw three displays that consisted of a task cue, followed by S1, and then S2. Both S1 and S2 consisted of a two-dimensional bar that varied in color and orientation. The task was to report the color or the orientation of S2 on the basis of the task cue. Both the task relevant and irrelevant features were independent. This led to S1 and S2 having a complete match in color and orientation on one-fourth of the trials, a partial match on two-fourths of the trials, and a complete mismatch on the rest of the trials. As S1 and S2 were identical on the complete match trials, they were likely to be perceived as different states of the same object, and this, in turn, should encourage spontaneous feature retrieval. If feature retrieval could occur with changing behavioral goals, participants would take longer to respond to S2 when S1 and S2 had a partial match compared to when they had a complete match or a complete mismatch.

### Methods

#### Ethics statement

This study received prior ethical approval from The University of Canterbury Human Ethics Committee. The committee approved the consent form and experimental procedure. Written consent was obtained from the participants.

#### Participants

Eighteen undergraduate students from the University of Canterbury volunteered for the experiment either in exchange for course credit or for payment. All of them reported having normal or corrected-to-normal vision.

#### Apparatus and stimuli

All stimuli were displayed against a grey background. They were shown on a Power Macintosh 6100/66 computer with a 13-in. RGB monitor. Participants were tested individually in a dimly lit room. The viewing distance from the monitor was approximately 60 cm. MacProbe [15] was used to generate stimuli and collect responses.

**Figure 1:**
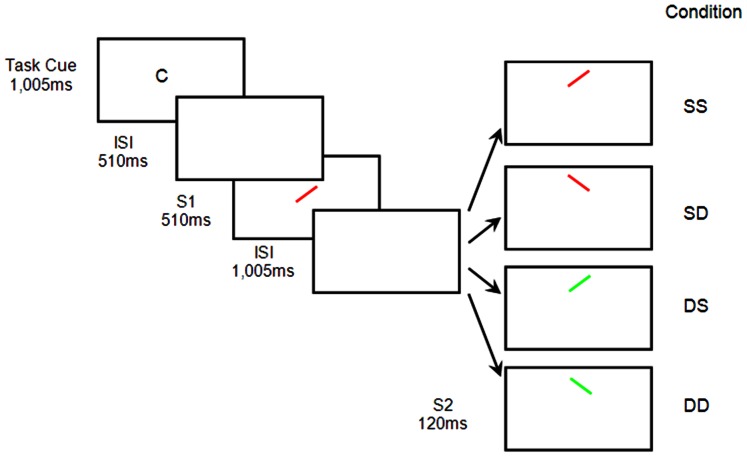
Examples of stimulus displays from Experiment 1. The task was to respond to the color (red or green) or orientation (left or right) of S2 as specified by a task cue, with a C referring to color and an O to orientation. Both the relevant and irrelevant features of S1 and S2 were independent. The figure depicts an example of a color task, in which color was a task relevant feature and orientation a task irrelevant feature. In this example, S1 and S2 had the same color and orientation in the SS condition, the same color but different orientation in the SD condition, the different color but same orientation in the DS condition, and different color and orientation in the DD condition.

Each trial started with a task cue, which was either a black letter C (for color) or O (for orientation) written in 36-point Geneva font at the center of a computer screen (see Figure 1). The cue was then followed by two successive displays, each consisting of a red or a green bar with a 45° left or right tilt from vertical. The bar subtended 0.57° of visual angle in length and 0.14° in width. To minimize masking, the two stimuli were shown at different locations. Whereas S1 always appeared at the center, S2 was either 4.12° above or below the center with equal probability.

#### Design and procedure

The experiment used a within-participants design. The principal manipulations were task (color vs. orientation), the relationship between the task relevant features of S1 and S2 (same vs. different), and the relationship between their task irrelevant features (same vs. different). Altogether, there were eight experimental conditions, four associated with the colour task, and the other four with the orientation task. The four conditions in each task were: the relevant-same- irrelevant-same (SS) condition, where S1 and S2 had the same relevant and irrelevant features; the relevant-same-irrelevant-different (SD) condition, where S1 and S2 had the same relevant but different irrelevant features; the relevant-different-irrelevant-same (DS) condition, where S1 and S2 had different relevant but same irrelevant features; and the relevant-different-irrelevant-different (DD) condition, where S1 and S2 differed in both the relevant and irrelevant features.

Each trial began with the presentation of the task cue for 1,005 ms, with a C referring to color and an O to orientation. The two types of trials were equally likely to appear, and they were randomly mixed within a block. After an inter-stimulus-interval (ISI) of 510 ms, S1 was displayed for 510 ms. Upon its offset, and followed by another ISI of 1,005 ms, S2 was shown for 120 ms. Participants were instructed to respond to S2 as quickly and as accurately as possible.

Participants were informed that S1 did not predict S2 in any way. They pressed one of four designated response keys on each trial, using their right index and middle fingers for the color task (with “.” for red and “/” for green) and their left middle and index fingers for the orientation task (with “z” for left and “x” for right). The experiment consisted of 48 practice trials, followed by four blocks of 80 trials. The entire experiment took approximately 40 minutes to complete.

### Results and Discussion

**Figure 2:**
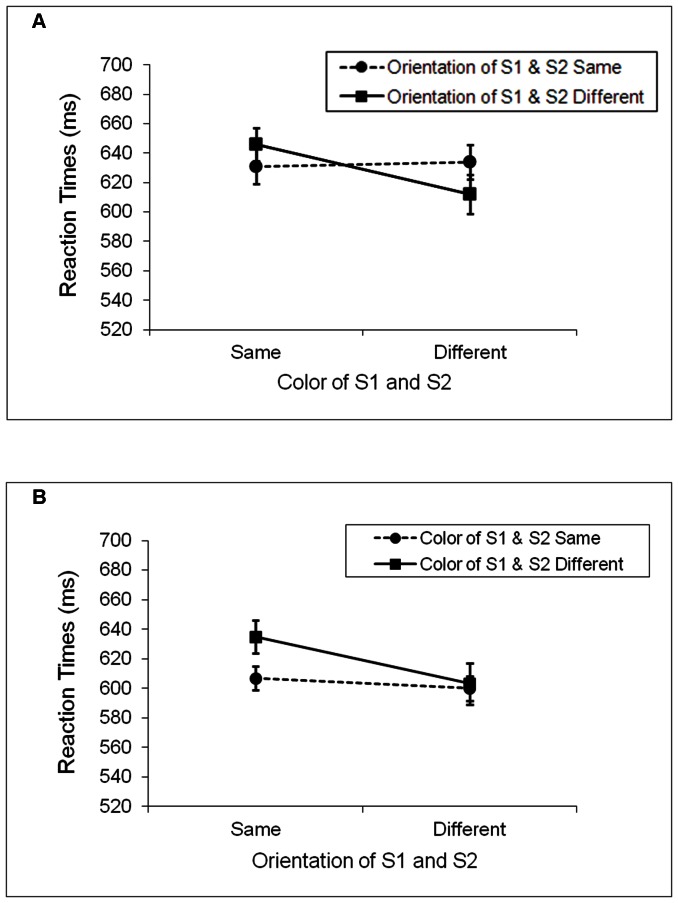
Mean reaction times for Experiment 1. A. The color task. B. The orientation task. Partial repetition costs were evident in the color task, but not in the orientation task.

Figures 2A and 2B show the mean RTs for the correct responses on the color and orientation trials, respectively. Reaction times greater than 2,000 ms were excluded from analyses. Such times accounted for less than1.2% of the total data both in this and the next two experiments. The mean error rates are shown in Table 1. The data from one participant were excluded from analyses due to long RTs (15% of the data were over the cutoff limit of 2,000 ms). Two separate 2×2×2 repeated-measures analyses of variance (ANOVAs) were conducted, one on accuracy and the other on RTs. The only significant result on accuracy was the main effect of task, *F*(1, 16) = 9.94, *MS_e_* = 75, *p*<.01. Participants were more accurate in the orientation task (5.8% error) than in the color task (10.5% error), suggesting that orientation discrimination was easier than color discrimination. No other significant effects were found.

**Table 1:** Mean Error Rates (Percent Incorrect) for Experiment 1.

	SS		SD		DS		DD	
Task	M	SE	M	SE	M	SE	M	SE
Color	9.1	1.2	11.2	1.7	10.0	0.9	11.9	1.2
Orientation	6.0	1.2	5.9	1.0	5.3	1.2	6.1	1.2

Note: SS: the relevant-same-irrelevant-same condition; SD: the relevant-same-irrelevant-different condition; DS: the relevant-different-irrelevant-same condition; and DD: the relevant-different-irrelevant-different condition.

With regard to RTs, ANOVA showed a significant main effect of relevant feature, *F*(1, 16) = 5.32, *MS_e_* = 1986, *p*<.05, indicating longer RTs when the relevant features of S1 and S2 matched (630 ms) rather than mismatched (612 ms). There was also a significant interaction between the relevant and irrelevant features, *F*(1, 16) = 4.84, *MS_e_* = 1680, *p*<.05, indicating partial-repetition costs. Specifically, when S1 and S2 had the same relevant feature, RTs were longer when the irrelevant features of the stimuli differed (641 ms) rather than when they matched (619 ms). Similarly, when S1 and S2 had different relevant features, RTs were longer when their irrelevant features matched (617 ms) compared to when they differed (608 ms). No other results were significant, and there was no evidence of speed-accuracy tradeoffs.

It is worth noting that the significant partial-repetition costs were driven primarily by the interaction between the relevant and irrelevant features on the color trials. Although there was no statistically significant 3-way interaction involving the type of task, visual inspection of the data revealed that the hallmark of partial-repetition costs, i.e., slower RTs when S1 and S2 had a partial match rather than a complete mismatch, was more evident in the color task. This suggests that the tightness of the binding between color and orientation may be influenced by which feature is the task relevant feature. We will return to this topic in the discussion of Experiment 2.

Another aspect of data that merits discussion was the finding of a significant main effect of relevant feature, i.e., RT was longer when the relevant features of S1 and S2 differed rather than when they matched. As S1 and S2 were independent, this result was puzzling. Inspection of the data revealed that this effect was caused largely by the shorter RT in the DD condition relative to the other conditions in the color task and the longer RT in the SD condition compared with the other conditions in the orientation task (see Figures 2A and 2B). A possible way to interpret the pattern of data for the color task is to take the locations of S1 and S2 into consideration even though we did not manipulate that in the experiment. Recall that S1 and S2 were always presented at different locations. If location played a role in the spontaneous feature retrieval between S1 and S2, then the SD and DS conditions, together with the SS condition, should all be considered as partial match conditions, and this would result in the observed longer RTs in these conditions than in the DD condition, where none of the features in S1 and S2 matched. As for why this pattern of data appeared only in the color task, but not in the orientation task, we have no good explanations at present.

The most important finding of the experiment is the partial-repetition costs, especially in the color task. Despite the fact that the task irrelevant feature of S1 and S2 remained relatively salient throughout the experiment, the participants still showed evidence of feature retrieval. These results suggest that spontaneous feature retrieval can occur with changing behavioral goals.

## Experiment 2

In Experiment 1, the relevant features of S1 and S2 were equally likely to be the same or different, and we found evidence for spontaneous retrieval of S1 features. In Experiment 2, we induced participants to inhibit S1 by matching the task relevant features of S1 and S2 on one-third of the trials, and mismatched them on the rest of the trials. As in Experiment 1, S1 and S2 were two-dimensional bars that varied in color and orientation, and their task irrelevant features were independent. This resulted in S1 and S2 being the same in color and orientation on one-sixth of the trials. Of specific interest was whether participants would again demonstrate partial-repetition costs. If such costs were found, this would indicate the spontaneous retrieval of features of S1 while S2 was being processed.

### Methods

The method was the same as that of Experiment 1 except for two differences. First, the task relevant features of S1 and S2 matched on one-third of the trials, and mismatched on two-thirds of the trials. Second, each of the four experimental blocks consisted of 120 trials, resulting in a total of 480 trials. The entire experiment took approximately 50 minutes to complete.

### Results and Discussion

**Figure 3:**
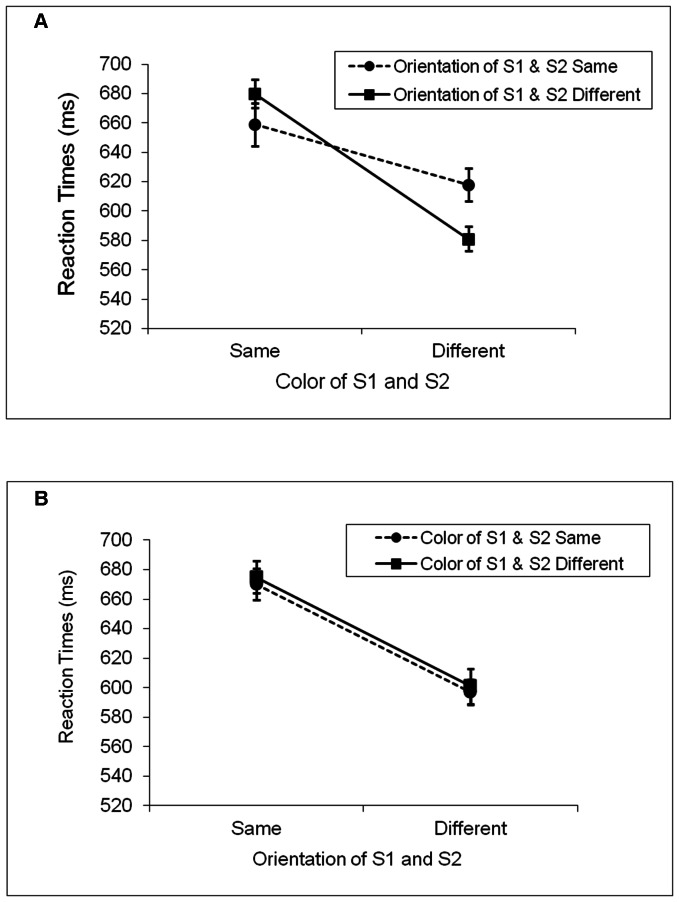
Mean reaction times for Experiment 2. A. The color task. B. The orientation task. Partial repetition costs were again found in the color task, but not in the orientation task.

Figures 3A and 3B show the mean RTs of correct responses on the color and orientation trials, respectively. A 2×2×2 repeated-measures ANOVA on mean RTs indicated that RT was longer when the relative features of S1 and S2 were the same (671 ms) than when they were different (599 ms), *F*(1, 23) = 29.52, *MS_e_* = 8417, *p*<.0001. There was also a significant 2-way interaction between the relevant and irrelevant features, *F*(1, 23) = 4.48, *MS_e_* = 2323, *p*<.05. A partial-repetition cost was found both when S1 and S2 had the same task relevant feature and when they had different task relevant features. Specifically, when S1 and S2 had the same relevant feature, RT was longer when their irrelevant features differed (i.e., a partial match between S1 and S2; RT = 678 ms) compared to when they matched (i.e., a match in both color and orientation between S1 and S2; RT = 664 ms). Similarly, when S1 and S2 had different relevant features, RTs were shorter when their irrelevant features also differed (i.e., a complete mismatch between S1 and S2; RT = 591 ms) relative to when they matched (i.e., a partial match between S1 and S2; RT = 607 ms). Finally, there was a significant 3-way interaction among task, relevant and irrelevant features, *F*(1, 23) = 5.83, *MS_e_* = 1683, *p*<.05. The last result suggests that the participants showed different patterns of data in the color and orientation tasks. No other effects reached significance.

To clarify the 3-way interaction, separate ANOVAs were performed on the color and orientation trials. On the color trials, RTs were faster when the colors of S1 and S2 were different (600 ms) relative to when they were the same (670 ms), *F*(1, 23) = 22.00, *MS_e_* = 5342, *p*<.001. There was also a significant interaction between the relevant and irrelevant features, *F*(1, 23) = 9.37, *MS_e_* = 2158, *p*<.01. Repeating the color of S1 in S2 impaired S2 responses when the orientation of S1 was changed (680 ms) relative to when its orientation was repeated (659 ms) in S2, *t*(23) = 1.73, *p*<.05. Furthermore, changing the color between S1 and S2 prolonged S2 responses when the orientation of S1 was repeated (618 ms) compared with when it was changed (581 ms) in S2, *t*(23) = 3.11, *p*<.01. The main effect of irrelevant feature was not significant, *F*(1, 23) <1, *n.s*.

On the orientation trials, the results were different. The only significant effect was the main effect of relevant feature, with faster RTs when the orientations of S1 and S2 were different (599 ms) relative to when they were the same (673 ms), *F*(1, 23) = 32.01, *MS_e_* = 4096, *p*<.0001. Neither the main effect of irrelevant feature nor the interaction between the relevant and irrelevant features was significant, *F*(1, 23) <1, *n.s.* in both cases. There was no evidence of partial-repetition costs.

The mean error rates are illustrated in Table 2. A 2×2×2 repeated-measures ANOVA on the accuracy data showed that the main effect of relevant feature was close to significance, *F*(1, 23) = 4.18, *MS_e_* = 14.17, *p* = .052. Consistent with the RT result, the error rate was lower when S1 and S2 had different relevant features (6.7%) than when they had the same relevant features (7.9%). There was also a significant interaction between the task and the relevant feature, *F*(1, 23) = 5.64, *MS_e_* = 13.61, *p*<.05. Whereas the relevant features of S1 and S2 matched or differed did not influence participants’ error rates in the color task (7.8% and 7.9% for the matched and mismatched trials, respectively), it affected the participants’ performance in the orientation task, with a higher error rate when the relevant features of S1 and S2 matched (8.0%) rather than differed (5.6%). No other effects reached significance. There was no evidence of speed-accuracy trade-offs.

**Table 2:** Mean Error Rates (Percent Incorrect) for Experiment 2.

	SS		SD		DS		DD	
Task	M	SE	M	SE	M	SE	M	SE
Color	7.6	1.1	7.9	0.9	7.4	0.7	8.3	0.7
Orientation	7.6	0.9	8.3	0.9	5.7	0.7	5.5	0.7

Note: SS: the relevant-same-irrelevant-same condition; SD: the relevant-same-irrelevant-different condition; DS: the relevant-different-irrelevant-same condition; and DD: the relevant-different-irrelevant-different condition.

Before we discuss the partial-repetition costs, it is necessary to establish that inhibition was evoked in Experiment 2. This is especially important given that repeating the relevant feature in S2 also led to longer RT in Experiment 1, where S1 and S2 were independent. One way to examine this issue is to conduct a cross-experiment analysis that compares the magnitude of the relevant feature effect in the two experiments. If inhibition was evoked in Experiment 2, the relevant feature effect should be larger in Experiment 2 than in Experiment 1.

We conducted a combined analysis on the RT data across the two experiments, using a mixed ANOVA with experiment as a between-subjects factor, and task, relevant and irrelevant features as within-subjects factors. For the sake of brevity, we report only the significant interactions with experiment, of which there was one. The interaction between the relevant feature and the experiment was highly significant, *F*(1, 39) = 10.16, *MS_e_* = 58719, *p* = .003. Whereas the difference in RT between the relevant-same and relevant-different trials was 72 ms in Experiment 2, it was 18 ms in Experiment 1. This result confirmed that the participants in the two experiments behaved differently when the relevant features of S1 and S2 matched vs. mismatched. Given the proportion of the relevant-same (one-third) vs. relevant-different (two-thrids) trials in Experiment 2, it seems reasonable to conclude that inhibition was evoked in Experiment 2.

To be cautious, we also examined the pattern of data in Experiment 2 while taking into account the fact that S1 and S2 were presented at different spatial locations. If we treat location as an important feature in Experiment 2, then the SS, SD, and DS conditions should all be considered as partial match conditions. A feature-retrieval-without-inhibition account would predict comparable RTs among these conditions. However, Tukey’s Honestly Significant Differences test showed that in both the color and orientation tasks, RT was significantly faster in the DS condition than in both the SS and SD conditions (*p*<.05 in all cases). This pattern of data is inconsistent with a feature-retrieval-without-inhibition account. Instead, it is consistent with the notion that inhibition was evoked in Experiment 2.

The most important finding of Experiment 2 was the partial-repetition costs in the color task. The participants took longer to respond to S2 when S1 and S2 differed in either color or orientation relative to when both features matched or mismatched. This pattern of data is similar to the results found in previous studies in which S1 was not inhibited ([3],[5],[9] and Experiment 1 in this study), suggesting that a similar mechanism may underlie the retrieval of features from a previously encountered object.

Partial-repetition costs were not found in the orientation task. While the exact nature of this null result was unclear, processing asymmetries between color and orientation have been reported in previous research [9],[16],[17]. In several experiments, Chen and Cave showed their participants stimulus displays that consisted of a target, a singleton distractor, and a homogenous group of other distractors. The task was to make a speeded color or orientation discrimination of the target on the basis of a task cue at the beginning of each trial. Responses to the target were either compatible or incompatible with a task irrelevant feature of the singleton distractor. The finding most relevant to the present experiment was that the effect of color on orientation differed from the effect of orientation on color. Whereas the orientation of the singleton had a reliable effect on the color of the target, the color of the singleton had negligible influence on the orientation of the target. A similar asymmetry between color and orientation was reported by Colzato et al. ([9] and Experiment 2), who manipulated the frequency of pairing between the different feature values in S2, and found a negligible effect of color on orientation when the conjunction between the specific feature values was infrequent. Interestingly, this null result was found only when S1 and S2 were simple geometric shapes such as bars, but not when they were bananas or strawberries. Huang et al. [17] also reported no effect of color on orientation judgment when color was a task irrelevant feature of a preceding target. These results suggest that although features are bound more or less spontaneously, the tightness of the binding and how each feature affects the processing of a subsequent stimulus may depend on a number of factors, including the nature of an individual feature attended and the long-term association between the relevant features in memory. We will return to this topic in the general discussion section.

## Experiment 3

The results of Experiment 2 show that the features of a recently inhibited object could be retrieved spontaneously when S1 and S2 were likely to be perceived as different instantiations of the same object. In Experiment 3, we changed the identity of S2 from a bar to a letter so that it was unlikely to be seen as a different state of S1. If the results of Experiment 2 were contingent upon the perceived object continuity between S1 and S2, the participants would show a different pattern of data in the present experiment. Moreover, the results of Experiment 3 should resemble the findings typically associated with object-based inhibition reported in previous studies [18]–[20].

A number of experiments have shown that when representation of S1 interferes with the processing of S2, participants sometimes demonstrate object-based inhibition, i.e., delayed responses to S2 when it shared features with S1 even though these features were irrelevant to the participants’ behavioral goals [18]–[20]. Importantly, even though perceived object continuity was not manipulated in these experiments, the specific S1 and S2 that were used were never identical, and were therefore unlikely to be seen as belonging to the same object. For example, Chao and Yeh [18],[19] investigated the effect of a task irrelevant prime (i.e., S1) on the color naming latencies of a probe (i.e., S2). Both the prime and probe consisted of a single Stroop color word, with the prime written in white ink and the probe written in colored ink incongruent with its meaning. When the meaning of the prime matched the color of the probe on a small proportion of trials within an experiment, probe RTs were longer on these “match” trials relative to the “nonmatch” trials where the two stimuli were unrelated. As meaning was a task irrelevant dimension, these results indicate object-based inhibition.

An important feature in the above-mentioned experiments is their use of the Stroop stimuli in both the prime and probe displays. With Stroop stimuli, the response codes between the relevant feature of the probe target (i.e., color) and the irrelevant feature of the prime distractor (i.e., meaning) were identical, making it unclear whether object-based inhibition would be evoked in experiments that use other visual stimuli.

In Experiment 3, S1 and S2 belonged to different types of stimuli. If features from S1 could only be spontaneously retrieved when object continuity was perceived between successive items, and if object-based inhibition could be generalized to non-Stroop stimuli, we should find slower RTs to S2 not only when S1 and S2 have the same rather than different relevant features, but also when they have the same rather than different irrelevant feature.

### Methods

The method was the same as that of Experiment 2 except for the identity of S2. To induce participants to see S1 and S2 as belonging to different objects, we changed the identity of S2 from a bar to a letter, making it a different type of object from S1. Instead of a colored bar, it was a colored letter: a V for some participants (N = 18), and a T for the other participants (N = 20). In both cases, the letter was written in 28-point Geneva font. Since bars and letters are different classes of objects, it was assumed that they would be seen as different entities. As in Experiment 2, the letter was tilted 45° to the left or right from the vertical, and the relevant features of S1 and S2 were the same on one-third of the trials, and different on the rest of the trials. Thirty-eight new volunteers from the same participant pool took part in the experiment.

### Results and Discussion

**Figure 4:**
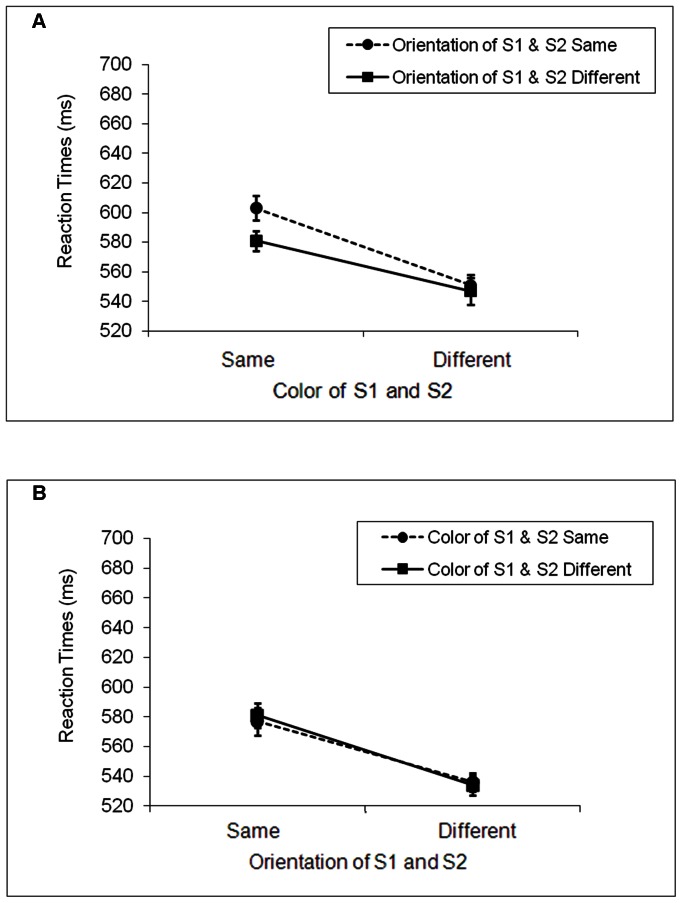
Mean reaction times for Experiment 3. A. The color task. B. The orientation task. There was no evidence of partial repetition costs in either the color or the orientation task. Note that the identities of S1 and S2 were always different, with S1 being a bar, and S2 a letter.

The data from two participants in the V group were excluded from analyses due to high error rates (>50% in multiple conditions). A four-factor (letter×task×relevant feature×irrelevant feature) repeated-measures ANOVA on participants’ mean RTs showed no significant effects concerning the specific letter used in the experiment, so the data from the two groups of participants were pooled in the rest of the analyses. The mean RTs for the color and orientation tasks are shown in Figures 4A and 4B, respectively. As in previous experiments, we performed a 2×2×2 repeated-measures ANOVA on participants’ mean RTs. Responses were again faster when the relevant features of S1 and S2 were different (542 ms) compared with when they were the same (586 ms), *F*(1, 35) = 29.97, *MS_e_* = 4617, *p*<.0001. Moreover, RTs were also faster when the irrelevant features of S1 and S2 were different (561 ms) rather than the same (567 ms), *F*(1, 35) = 4.27, *MS_e_* = 620, *p*<.05. The three-way interaction among task, relevant, and irrelevant features was also significant, *F*(1, 35) = 4.13, *MS_e_* = 617, *p* = .050. There were no other significant results.

The color and orientation trials were again analyzed separately. In the color task, both the relevant and irrelevant features affected performance. RTs were slower when S1 and S2 had the same relevant feature (592 ms) compared with different relevant features (549 ms), *F*(1, 35) = 23.13, *MS_e_* = 2895, *p*<.001. Importantly, unlike the results of Experiment 2, RTs were also slower when S1 and S2 had the same irrelevant feature (577 ms) relative to different irrelevant features (564 ms), *F*(1, 35) = 7.66, *MS_e_* = 762, *p*<.01. There was no significant interaction between the relevant and irrelevant features, *F*(1, 35) = 2.65, *MS_e_* = 1082, *p*>.10. These results provide no evidence for feature retrieval. Instead, they suggest that both the relevant and irrelevant features of S1 were inhibited in the color task.

The results in the orientation task were similar to those found in Experiment 2. Participants again showed evidence of inhibiting the relevant feature, with longer RTs when S1 and S2 had the same relevant feature (579 ms) than when they had different relevant features (535 ms), *F*(1, 35) = 26.33, *MS_e_* = 2713, *p*<.0001. Neither the main effect of the irrelevant feature nor the interaction between the relevant and irrelevant features was significant, *F*(1, 35) <1 in both cases. These results echoed those found in Experiment 2 in that while orientation appeared to influence color discrimination, color did not affect orientation judgment.

The error rates are shown in Table 3. Consistent with the RT data, error rates were higher when the relevant features of S1 and S2 matched (10.9%) rather than mismatched (8.2%), *F*(1, 35) = 5.78, *MS_e_* = 89, *p*<.05. However, there were also a significant main effect of irrelevant feature, *F*(1, 35) = 5.0, *MS_e_* = 21, *p*<.05, and a near significant interaction between task and relevant feature, *F*(1, 35) = 3.9, *MS_e_* = 24, *p* = .055. Subsequent analyses revealed that whereas there were no reliable main effects or an interaction in the color task, there were two significant main effects in the orientation task. Participants made more errors when the task relevant features of S1 and S2 matched (11.2%) rather than mismatched (7.4%), *F*(1, 35) = 6.98, *MS_e_* = 75, *p*<.05. Surprisingly, they also made more errors when the task irrelevant features of S1 and S2 differed (10.1%) compared with when they matched (8.5%), *F*(1, 35) = 5.46, *MS_e_* = 17, *p*<.05. The last result was unexpected, and we have no explanation for it. There was no significant interaction between the relevant and irrelevant features, *F*(1, 35) <.1, *n.s*.

**Table 3:** Mean Error Rates (Percent Incorrect) for Experiment 3.

	SS		SD		DS		DD	
Task	M	SE	M	SE	M	SE	M	SE
Color	10.5	0.9	10.8	0.9	8.4	0.8	9.8	0.8
Orientation	10.4	0.8	12.1	1.4	6.7	0.9	8.1	0.9

Note: SS: the relevant-same-irrelevant-same condition; SD: the relevant-same-irrelevant-different condition; DS: the relevant-different-irrelevant-same condition; and DD: the relevant-different-irrelevant-different condition.

To verify that the pattern of data in Experiments 2 and 3 differed significantly, we did a combined analysis on the RT data across the two experiments with experiment as a between-subjects factor, and task, relevant, and irrelevant features as within-subjects factors. Again, for the sake of brevity, we report only the significant interactions that involve experiment. Two effects were found. One was a significant 3-way interaction among experiment, relevant, and irrelevant features, *F*(1, 58) = 5.57, *MS_e_* = 9099, *p* = .02. The second was a significant 4-way interaction, *F*(1, 58) = 11.38, *MS_e_* = 11711, *p* = .001. Subsequent analyses on the color and orientation trials separately indicated that the 4-way interaction arose primarily from the participants in the two experiments behaving differently in the color task, where a significant 3-way interaction of experiment, relevant, and irrelevant features was found, *F*(1, 58) = 13.74, *MS_e_* = 20728, *p* = .001. A similar 3-way interaction was not found in the orientation tasks, *F*(1, 58) <1, *ns*. These results confirmed that the pattern of data in Experiments 2 and 3 differed in the color task. Thus, unlike the participants in Experiment 2, who showed a crossover interaction between the relevant and irrelevant features of S1 and S2 on the color trials (i.e., the partial-repetition costs that were indicative of feature retrieval from S1), the participants in Experiment 3 did not show a crossover interaction. Instead, they showed main effects of the relevant and irrelevant features, with slower RTs both when the task relevant features of S1 and S2 matched rather than mismatched, and when the task irrelevant features of S1 and S2 matched rather than mismatched. These results are consistent with the object-based inhibition observed in previous studies where S1 and S2 were also different [18]–[20].

However, despite a significant main effect of irrelevant feature in the color task, one may notice that the effect was driven largely by the differential RTs between the SS and SD conditions (a difference of 22 ms) instead of between the DS and DD conditions (a difference of 4 ms). How can we explain this pattern of data?

If we believe that attention is object-based in addition to location-based ([8],[21]–[24]; also see [25] for a review) and that attending to a stimulus causes the integration of the features that belong to the attended stimulus [26]–[28], then the above-mentioned different pattern of data can be explained in the following way. Let us suppose that on a given color trial, S1 was a red bar with a left orientation. As “red” and “left” belonged to the same object, the two features were associated and were both inhibited. When the relevant features of S1 and S2 were identical, S2 would be a red letter with a left orientation in the SS condition, and a red letter with a right orientation in the SD condition. Because “red” was associated with “left” but not with “right”, responses would be delayed when S2 was red and had a left orientation in the SS condition, compared with a right orientation in the SD condition. Similar reasoning can be applied to those trials in which the relevant features of S1 and S2 were different. If we again suppose that S1 was a red bar with a left orientation, S2 would be a green letter with a left orientation in the DS condition and a green letter with a right orientation in the DD condition. As the inhibited “left” was not associated with “green”, responses to S2 (whose color was green) would be independent of its specific orientation, resulting in comparable RTs between the DS and the DD conditions. Thus, our data are consistent with an object-based inhibition of S1 even though there was no appreciative difference in RTs between the DS and DD condition.

The results of Experiment 3 indicate that spontaneous feature retrieval between S1 and S2 is influenced by perceived object continuity. However, a different conclusion was reached in a recent study by van Dam and Hommel [29]. In a series of experiments (Experiments 1 through 4), the participants in van Dam and Hommel were shown two sequentially presented displays (S1 and S2), each consisted of a red or green circular object containing a smaller yellow elliptic object that was either vertically or horizontally orientated. The task was to determine the orientation of the elliptic object in S2. The principal manipulations were the color of the circular object and the orientation of the elliptic object between S1 and S2. S1 and S2 could be completely matched (i.e., same color and orientation), partially matched (i.e., same color but different orientation or vice versa), or completely mismatched (i.e., different color and orientation). In addition to these within-experiment manipulations, the authors also systematically varied the relationship between the circular and elliptic objects so that from Experiment 1 through 4, these objects were increasingly more likely to be perceived as two distinct objects (e.g., an apple partly occluded by a banana in Experiment 4) rather than as a single object (e.g., a colored ball with a horizontal or vertical stripe in Experiment 1). Partial repetition cost, which was calculated as the mean of color repeated and orientation repeated trials minus the mean of the neither repeated and both repeated trials, was found in all the four experiments. Furthermore, the magnitude of the cost was comparable across the experiments. Based on these results, the authors concluded that object cues are unlikely to be relevant for the retrieval of features in visual perception.

It is interesting to note that although the conclusion reached by van Dam and Hommel [29] was different from the conclusion we reached in the present study, a careful examination of their data (Table 1, p. 1189) revealed that their conclusion was based largely on the way the partial repetition cost was calculated. If we compare the reaction times of those trials in which orientation, i.e., the task relevant feature, was repeated from S1 and S2 (a partial match) with those trials in which neither orientation nor color was repeated from S1 and S2 (a complete mismatch), there was a systematic decrease in the magnitude of the partial repetition cost from Experiment 1 to Experiment 4 (the partial repetition costs were: 19 ms, 9 ms, 5 ms, and −6 ms from Experiments 1 to 4, respectively). As the stimuli from Experiment 1 to Experiment 4 were increasingly more likely to be perceived as two distinct objects rather than as a single object, these results suggest that perceived object continuity can influence, at least to some degree, the retrieval of features between two successive stimuli.

## General Discussion

Previous work has established that when two objects are in close spatiotemporal sequence, attention to an object can trigger the retrieval of features of a previously viewed object [3],[5],[6]. In the present experiments, we generalized this finding to an object that had recently been actively inhibited. Experiments 1 and 2 showed that when two successively presented stimuli could be seen as different states of the same object, attention to S2 would trigger the retrieval of features from S1, regardless of whether S1 and S2 were independent (Experiment 1), or whether S1 was known to be different from S2 on a majority of trials (Experiment 2). Experiment 3 demonstrated that inducing participants to see S1 and S2 as different objects could disrupt the feature retrieval process, resulting in performance consistent with object-based inhibition. Of course, because we used letters as S2 in Experiment 3, and the processing of letters may differ from that of other visual stimuli in non-trivial ways, it is unclear to what degree the present results were caused by the specific stimuli used in our experiments. Future research is needed to explore this issue.

### Feature Retrieval and Object-Based Inhibition

Although we explained the results of Experiment 2 in terms of spontaneous feature retrieval and the results of Experiment 3 in terms of object-based inhibition, we do not believe that these two mechanisms are mutually exclusive. In fact, we consider it likely that object-based inhibition was also evoked in Experiment 2, and that the partial-repetition costs found in the color task were the results of competition between the two mechanisms. Evidence for the spontaneous feature retrieval account comes mainly from the slower RT in the SD condition than in the SS condition. However, if reactivating an inhibited object took less time than resolving the conflicting codes associated with S1 and S2, RTs would be faster when S1 and S2 matched in both color and orientation in the SS condition relative to when only one of these features was matched in the SD condition, even though object-based inhibition had been deployed (i.e., both the relevant and irrelevant features of S1 were inhibited). Thus, the lack of behavioral manifestation of object-based inhibition does not necessarily imply that the latter was not evoked. The effect of object-based inhibition could be masked by a fast-acting feature retrieval process. The idea that participants could inhibit an object under some circumstances with no measurable inhibitory effects is consistent with the results of a recent study by Wyatt and Machado [30]. They manipulated the response compatibility and stimulus-onset-asynchrony (SOA) between the target and distractors (with the onset of the distractors always preceding that of the target), and found evidence for distractor inhibition even though their participants showed no significant negative compatibility effect when the distractor-target SOA was long.

The notion that multiple mechanisms can co-exist within a single paradigm is not new. It has been shown in experiments on object-based attention (see [25] for a review) and in experiments that used the inhibition of return (IOR) paradigm [31]–[36]. In both types of experiments, attention is found to select both location and object, either in the form of facilitation (in object-based attention studies) or inhibition (in object-based IOR), suggesting that multiple mechanisms can exist within a single task.

### The Role of Location in Feature Retrieval

In their seminal paper on the object file theory, Kahneman et al. [6] stressed the importance of location in the retrieval of features of a previously viewed object. Their participants (in Study 3) showed evidence for feature retrieval when a spatial correspondence could be found between S1 and S2 via apparent motion. In contrast, a similar effect was not found when the locations of S1 and S2 across different frames did not give rise to the perception that S1 and S2 were different states of the same object. These results led the authors to the conclusion that object files are addressed in location instead of in non-spatial object features such as color or form. Evidence that supports the crucial role of location in the retrieval of features has also been reported by Mitroff and Alzarez [7] and Saiki [37].

The unique role of location proposed in the object file theory is in line with many theories of attention that emphasize the importance of location in selective attention [38]–[46]. There is considerable evidence that attending to an object feature results in the selection of its location regardless of participants’ behavioural goals ([47]–[50], see also [51] for a review). In contrast, attending to an object’s location does not lead to the encoding of an object feature such as color, shape, or texture when the feature in question is not task relevant [48],[50]. These results suggest the unique role of location in visual attention.

In the present study, our participants showed evidence for episodic feature retrieval in Experiments 1 and 2 despite the fact that S2 was always at a different spatial location from S1. However, this does not mean that our result is contradictory to what was found in prior research. An important methodological difference between the present experiments and prior research [6],[7],[37] is the number of objects in the S1 display. Whereas a single object was used in the S1 display in Experiments 1 and 2 of the present study and in the other experiments that found evidence for episodic feature retrieval via non-spatial object features [3],[5],[9], multiple objects were used in the studies that showed the essential role of location in successful feature retrieval [6],[7],[37]. When multiple objects are present in the S1 display, there is uncertainty over the relationship between S2 and a specific S1, and spatial correspondence may be essential in establishing perceived object continuity between two successive stimuli. In contrast, when a single object appears in two sequential displays, the visual system may have a natural tendency to link them regardless of the difference in locations, so long as there is a reasonable match in other features. Thus, even though a location match did not appear to be required in some experiments ([3],[5],[9] and Experiments 1 and 2 of the present study), this by no means suggests that a spatiotemporal correspondence was not established in these studies.

### Feature Asymmetry and S1 Inhibition

In the experiments reported here, there was a feature asymmetry between color and orientation. Whereas the color of S1 had a clear effect on the orientation judgement of S2, the orientation of S1 had a negligible influence on the color discrimination of S2. One possible explanation of this asymmetry is that in comparison with orientation, color may be a relatively difficult feature to inhibit, and may therefore take longer to be suppressed when it is a task irrelevant dimension. After all, under most circumstances, we do not expect an object to change color, but we do expect an object to change orientations. A book placed vertically on a bookshelf is likely to be seen as the same book when it is lying horizontally. However, a book with a red cover is unlikely to be seen as the same book when its cover becomes green.

If color is a relatively difficult irrelevant feature to inhibit when it is paired with orientation, the null result of color on orientation should disappear when facilitation rather than inhibition is applied to S1. This was indeed what was found in two experiments by Chen [52], in which the relevant features of S1 and S2 matched on two-thirds of the trials, and mismatched on one-third of the trials. In both experiments, when S1 and S2 had the same relevant features, the irrelevant feature of S1 influenced the processing efficiency of S2 regardless of whether the task was color or orientation. In other words, having the same orientation between S1 and S2 facilitated the color judgment of S2. Similarly, having the same color between S1 and S2 also speeded up the orientation judgment of S2. There was no asymmetry between color and orientation when participants were encouraged to maintain an active representation of S2. Although these results do not address directly the question of the underlying cause of the asymmetry observed in the experiments reported here, they are consistent with our conjecture that the asymmetry could be due to the differential amount of time required to suppress color and orientation when they are features of the same objects. This interpretation is also consistent with the results of Chen and Cave [16].

To conclude, the present experiments suggest that the features of a previously inhibited object can be retrieved spontaneously. However, such retrieval and its effect on a subsequent target depend on the perceived object continuity between the two successive stimuli.
